# Data on a novel approach examining the role of the cerebellum in gait performance improvement in patients with Parkinson disease receiving neurologic music therapy

**DOI:** 10.1016/j.dib.2023.109013

**Published:** 2023-02-27

**Authors:** Antonino Naro, Loris Pignolo, Daniele Bruschetta, Rocco Salvatore Calabrò

**Affiliations:** a IRCCS Centro Neurolesi Bonino Pulejo, Messina, Italy; b Istituto S. Anna, Crotone, Italy; c Azienda Ospedaliera Universitaria Policlinico G.Martino, Messina, Italy

**Keywords:** Gait rehabilitation, Parkinson's disease, Rhythmic auditory stimulation, Cerebellum

## Abstract

Individuals with idiopathic Parkinson's disease (PD) benefit from Rhythmic Auditory Stimulation (RAS) concerning gait impairment recovery. In PD, RAS may help eliciting rhythmic and automatized motor responses, including gait, by bypassing the deteriorated internal “clock” within basal ganglia for automatic and rhythmic motricity. We aimed at exploring the contribution of the cerebellum to this “bypass effect” in response to RAS. To this end, we examined the cerebellum-cerebral connectivity indices using conventional EEG recording to assess whether the cerebellum contributes to RAS-based post-training effects in persons with PD. Fifty PD patients were randomly assigned to an 8-week training program using Gait-Trainer3 with or without RAS. We measured the Functional Gait Assessment, the Unified Parkinson's Disease Rating Scale, the Berg Balance Scale, the Tinetti Falls Efficacy Scale, the 10-meter walking test, the timed up-and-go test, and the gait quality index derived from gait analysis before and after the end of the training. A standard EEG during gait on the GT3 was also recorded and submitted to eLORETA analysis. Particularly, we focused on the time course of the gait-related activities (which were characterized using the maximum amplitude vertex across the gait cycles) within each brain region of interest. These clinical and electrophysiological measures could be used to monitor the improvement in gait performance in standard clinical settings and to develop new rehabilitation protocols focusing on a holistic functional recovery approach.

**Specifications Table**

**Table:** 

Subject	Physical Therapy and Rehabilitation
Specific subject area	Electrophysiological assessment of robot-aided gait training (RAGT) post-training effects for the recovery of motor functions in Parkinson's disease (PD) patients
Type of data	Excel file for clinical and EEG data
How the data were acquired	Data were acquired using standardized clinical measures, including the Functional Gait Assessment (FGA), the Unified Parkinson's Disease Rating Scale (UPDRS), the Berg Balance Scale (BBS), the Tinetti Falls Efficacy Scale (FES), the 10-meter walking test (10MWT), the timed up-and-go test (TUG), and the gait quality index (GQI) derived from gait analysis. EEG was recorded using a Brain-Quick System (Micromed; Mogliano Veneto, Italy) equipped with a standard 21-electrode headset (Fp1,Fp2,F7,F3,Fz,F4,F8,T3,C3,Cz,C4,T4,T5,P3,Pz,P4,T6,O1,O2, plus a reference- and a ground-electrode). EEG was sampled at 512Hz, band-pass filtered at 1-200Hz using a zero-phase 36-7500 finite impulse response (FIR) filter, and notch-filtered at 50Hz (FIR notch filter, order=3302) to remove the power line noise. A common average served as reference for the purpose of source imaging analysis. Impedance was always below 5kΩ. A bipolar electro-oculogram (EOG) was also collected.
Data format	Raw clinical data Analyzed EEG data
Description of data collection	Clinical outcome data were collected by blinded clinicians before and after the gait training completion. EEG was recorded during the entire gait practice at a full step pace (i.e., 20 minutes), in order to obtain as many trials as possible, because signal to noise ratio in cerebellar EEG recordings is a challenge because of distance of electrodes to neural signal. Through capturing a whole stride, continuous data was divided into epochs extending from the proper heel strike (HS) to the next one (composed of the subsequent, in order: right, left, and right HS). We thus obtained 428±25 epochs. Gait cycle phases were indexed by a wireless inertial sensor (GSensor, BTS Bioengineering; Milan, Italy) fixed on the patient's torso using Velcro straps and used to trigger the EEG. Then, Data processed using EEGLab to remove artefactual epochs and components (by visual inspection and Infomax algorithm Independent Component Analysis(ICA)) [1]. The time-points of the epochs for the HS were therefore realigned for the trimmed, segmented EEG, with the correct, left, and right HS time-warped to 0, 50, and 100 percent of the gait cycle, respectively. [2]. Source imaging analysis was performed on the epoched EEG using eLORETA plugin into EEGLab [3]. The head model for source imaging analysis across all subjects was based on the standard Montreal Neurological Institute coordinates (MNI)-152 template anatomy, adding the cerebellar parcellations in nonlinear MNI-152 space, and finally merging the cortical and cerebellar surface models. The current dipoles were placed at the vertices of the cerebellar and cortical surface models and limited to the orientations orthogonal to their respective surfaces, resulting in improved source localization spatial focus. The combined brain and cerebellar model was used to perform source imaging using a weighted-minimum norm estimate [4,5]. The current dipole amplitudes were then vertex-wise normalized to the baseline and utilized to create source maps with
	z-score values for each 25% of the gait cycle and subject, which were then averaged across subjects. Gait-relevant cortical regions of interest (ROIs) were defined in an electrode-based approach (left and right frontal, central, parietal, temporal, and occipital ROIs). Gait-relevant cerebellar ROIs were defined on the largest cerebellar peaks of activations. The time course of the gait-related activity within each ROI was characterized using the maximum amplitude vertex across the gait cycles.
Data source location	Institution: IRCCS Centro Neurolesi Bonino Pulejo City/Town/Region: Messina Country: Italy Latitude and longitude (and GPS coordinates, if possible) for collected samples/data: latitude: 38.205282; longitude: 15.519191; GPS 38°12’21.698”, 15°31’9.321”
Data accessibility	Repository name: “EEG data repository” on Mendeley Data. Data identification number (permanent identifier, i.e. DOI number): 10.17632/yrfkgrwtg5.1. Direct link to the dataset: https://data.mendeley.com/datasets/yrfkgrwtg5/1 Repository name: “clinical data repository” on Mendeley Data. Data identification number (permanent identifier, i.e. DOI number): 10.17632/pb6 × 8dt9cr.1. Direct link to the dataset: https://data.mendeley.com/datasets/pb6×8dt9cr/1
Related research article	Naro A, Pignolo L, Bruschetta D, Calabrò RS. What about the role of the cerebellum in music-associated functional recovery? A secondary EEG analysis of a randomized clinical trial in patients with Parkinson disease. Parkinsonism Relat. Disord. 2022; 96:57-64.

## Value of the Data

• Gait training with RAS provided PD patients with a greater gait performance improvement than the gait training without RAS did. Such a gait improvement is likely to be related to a RAS-induced potentiation of the cerebellum-cerebrum connectivity that compensates for the impaired cortico-basal ganglia circuitry related to gait performance.
• The findings could be used as a point of comparison with studies evaluating the improvement in gait performance in PD patients and pave the way for further trials deeply assessing the role of cerebellum-cerebrum connectivity improvement related to gait training.
• The information might be utilized to build more tests for the validation of additional electromedical and robotic neurorehabilitation devices. Furthermore, detecting an impaired cerebellum-cerebral connectivity may help identifying the patients whose motor program must include cerebellar function training in order to improve gait.

## 1 Data Description

Gait impairment in Parkinson's disease (PD) is characterized, among other, by an irregularity of walking pace, reduced stride length and step velocity, and increased cadence, and freezing of gait [6]. These alterations have a common denominator in the difficulty in performing automatized movements, including walking [7]. The impairment in performing automatized movements mainly depends on the dopaminergic output failure within basal ganglia [8], [9], [10]. Beyond the latter, the role of the cerebellum in gait abnormalities in PD has been deeply investigated, also in view of the potential application in the rehabilitation field. The cerebellum may be involved in regulating prediction and movement sequencing [11,12] and may compensate for the basal ganglia impairment [13], [14], [15], thus having a significant role in regulating gait sequencing output, which is critically impaired in PD. Based on this premise, we aimed at assessing the contribution of the cerebellum to externally paced gait training using rhythmic auditory stimulation (RAS) in PD patients. To this end, we explored the cerebellum-cerebral EEG connectivity changes, seeking whether RAS influences such a connectivity through the above mentioned neural systems controlling motor performance.

### 1.1 Clinical data before the training

**Fig. 1:**
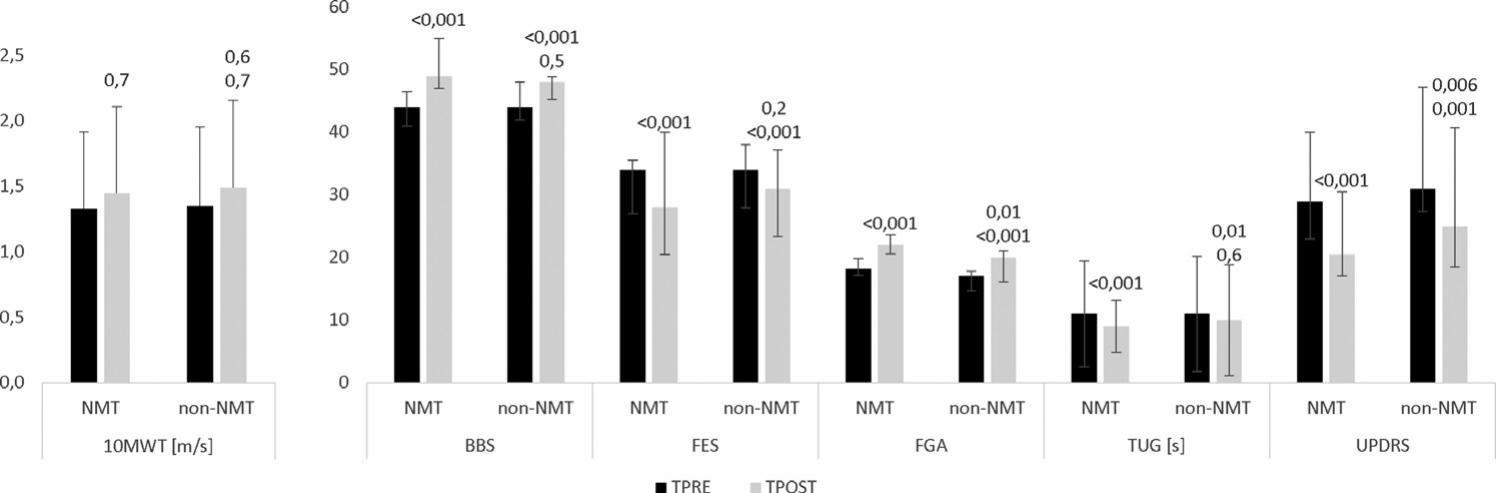
Effects of gait training on clinical parameters before (TPRE) and after the end of the training (TPOST). Data are reported as mean/median and standard deviation/interquartile range (vertical error bars). Superscript numbers represent the p-values of within-group and between-group (#) changes

The clinical data (available at https://data.mendeley.com/datasets/pb6 × 8dt9cr/1) refer to the effectiveness of gait training with RAS (NMT group) and without RAS (non-NMT group). The clinical parameters were obtained at the start (before the training) and at the conclusion (after the end of the training) of the rehabilitation period. Between the two groups, there were no significant clinical-demographic differences (Fig. 1). Additionally, there were no significant differences in gait and balance tasks and in overground gait performance (as per GQI) between the groups. In fact, both groups had a low GQI. Patients showed a modest range of disability, which was similar in both groups.

### 1.2 Clinical data after the end of the training

Both trainings yielded a significant improvement in FES (both p<0.001), FGA (both p<0.001), and UPDRS (both p<0.001); the changes were of greater magnitude in the NMT group than in the non-NMT group concerning FGA (p=0.01) and UPDRS (p=0.006) (Fig. 1). Conversely, the groups equally improved in FES (p=0.2), BBS (p=0.5), and TUG (p=0.6). The 10MWT only slightly improved in both groups (Fig. 1).

### 1.3 EEG data before the training

**Fig. 2:**
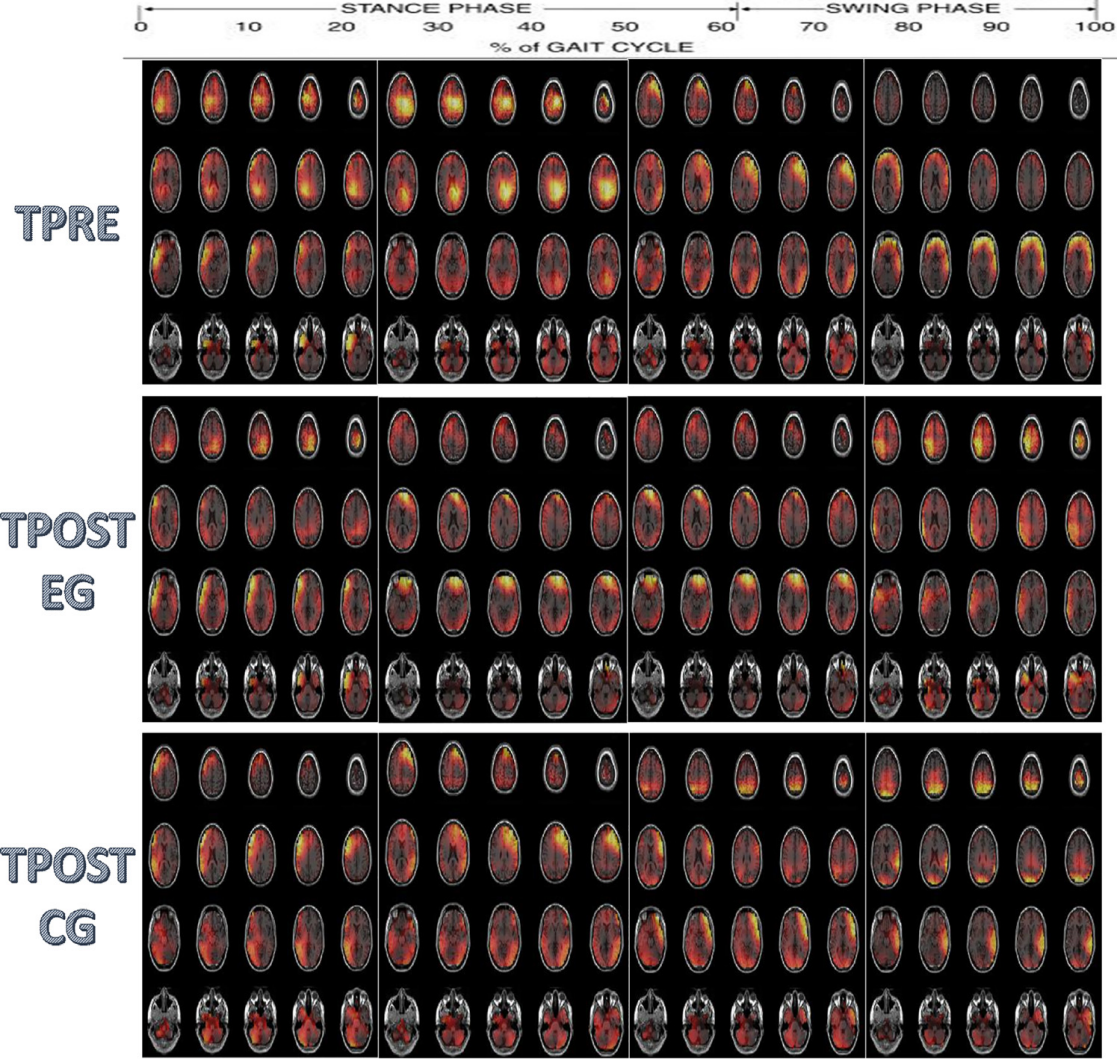
Group-average source maps of spatiotemporal patterns of activity with z-score values at each of the gait cycle phases (0-25, 25-50, 50-75, and 75-100%), group (EG and CG), and assessment (before (TPRE) and after the end of the training (TPOST)).

The EEG data (available at https://data.mendeley.com/datasets/yrfkgrwtg5/1) refer to the electrophysiological correlation of the clinical improvement induced by the gait training. Before the training (Fig. 2), in both groups, there was a significant gait-related activation of left sensorimotor cortices (0-25% of the gait cycle, including BA4-paracentral lobule and BA1), i.e., contralateral to the right-HS and during the swing-phase of the left limb, which became bilateral in the 25-75% of the gait cycle (i.e., during the left limb swing completion and the initiation or the right limb swing up to the mid-swing), and it turned back contralateral to the left HS (75-100% of the gait cycle, i.e., the left swing completion). During the entire gait cycle, a high activation of both the frontal areas, including BA6 (Secondary Motor Cortex -PMC and SMA), BA9 (dorsolateral-PFC), and BA10 (anterior-PFC) was also appreciable. Lastly, a bilateral, significant cerebellar lobe activity (mainly on lobule VI-Crus I) was observed in the 25-75% of the gait cycle.

### 1.4 EEG data after the end of the training

**Fig. 3:**
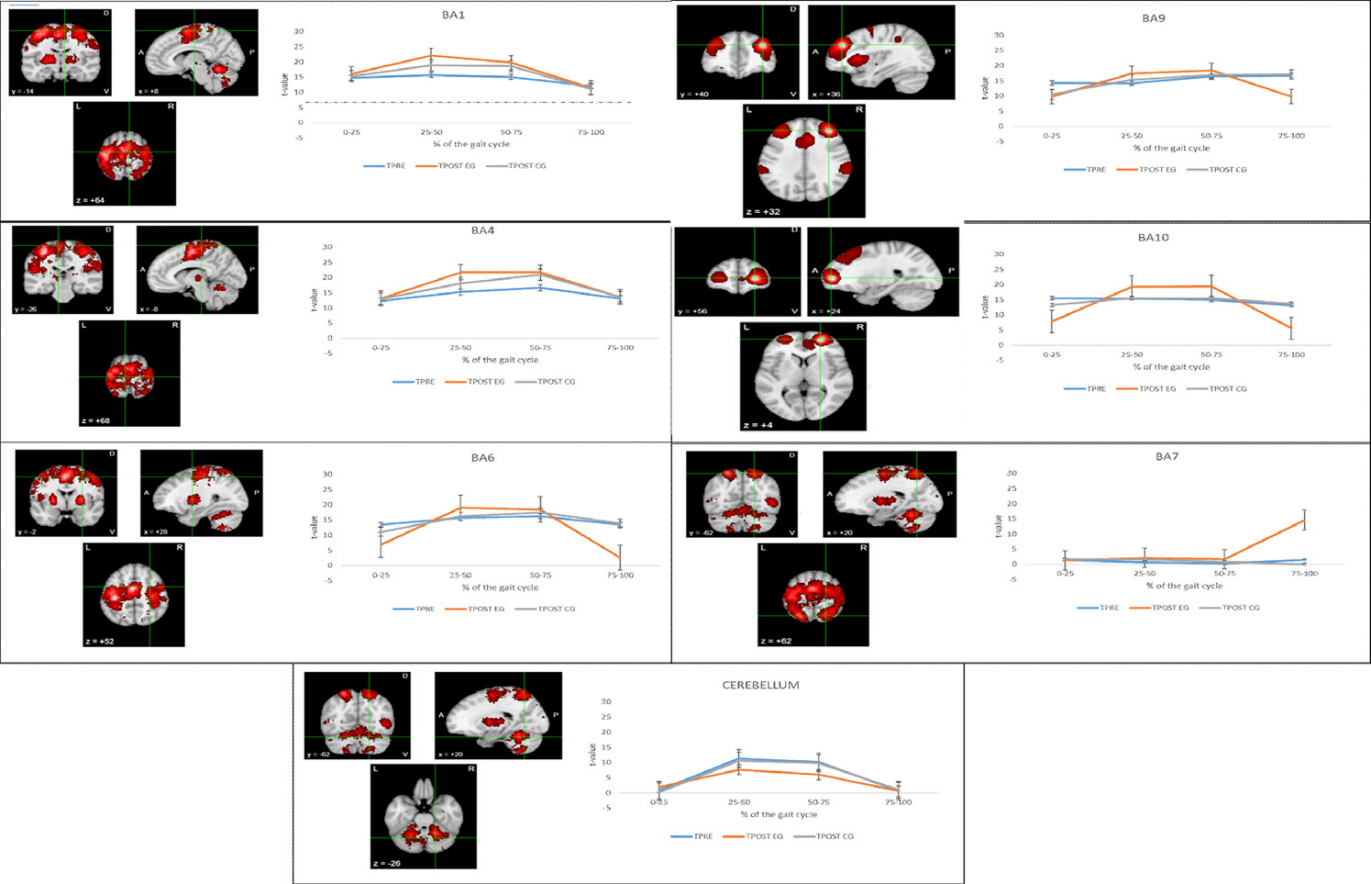
The time course of the local maxima of the regions showing functional activation (t-value, thresholded at corrected p<0.0002 –dotted line) at each of the gait cycle phases (0-25, 25-50, 50-75, and 75-100%), group (EG and CG), and assessment (before (TPRE) and after the end of the training (TPOST)). Activity is shown for the maximum amplitude vertex within each ROI during the gait epoch.

After the end of the training in the NMT group, we observed a reduction of the frontal area activation that was observed before the training in both the 0-25% and 25-75% of the gait-cycle (both p<0.0001) (Fig. 2, Fig. 3). Moreover, we found a potentiation of the activation within both the central and parietal areas in the 25-75% of the gait-cycle (p<0.0001). In addition, an activation of the parietal and occipital areas in the 75-100% of the gait-cycle was appreciable instead of the frontal areas (p=0.0001) (Fig. 1, Fig. 3). Lastly, there was a reduction of cerebellar activity in the 25-50% (approximately on the more lateral portions) (Fig. 1; p<0.0001) and in the 50-75% of the gait-cycle (approximately on the posterior cerebellum) (Fig. 3; p<0.0001).

After the end of the training in the non-NMT group, we observed a significant reduction of the frontal activation observed before the training along the entire gait-cycle (p=0.003); however, such a reduction was smaller than that found in the NMT group (between-group difference p=0.001) (Fig. 2, Fig. 3). There was also a potentiation of the centroparietal areas activation in the 25-75% of the gait-cycle observed before the training (p=0.002); however, such a potentiation was milder than that observed in the NMT group (between-group difference p<0.001). Furthermore, it lacked the gait-cycle specificity observed in the NMT group (Fig. 2, Fig. 3). Finally, the cerebellar activation we observed at baseline did not show any significant change after the end of the training (p=0.3), contrarily to what occurred in the NMT group (between-group difference p<0.0001) (Fig. 2, Fig. 3).

## 2 Experimental Design, Materials and Methods

### 2.1 Population and trial design

Patients were enrolled in this trial, among those attending our Rehabilitation and Research Institute as in-patient, if they met the following criteria: (i) Hoehn and Yahr stages II and III, Mini-Mental State Examination test >23, and normal executive function tests; and (ii) no modifications in antiparkinsonian medication treatment in the past 6 months. A history of neoplasms, severe cardiovascular, pulmonary, visual, auditory, and muscular-skeletal illness, other neurological diseases, and neurologic music treatment in the previous three months were all considered exclusion criteria. Fifty participants were included in the trial and 1:1 randomized in either the RAS treadmill gait training (NMT group) or the non-RAS treadmill gait training (non-NMT group). Before being enrolled, all participants gave written informed consent to participate in the trial and have their data published. Patients belonging to the NMT (n=25) were provided with a daily, 30-minute session of treadmill gait training using the Gait-Trainer3 (GT3) (Biodex; Shirley, NY, US), 5 days weekly, for two months. The patients belonging to the non-NMT (n=25, homogeneous for clinical and demographic features) were provided with an equal amount of GT3 gait training without using RAS. Regardless of group assignment, all patients received in addition a daily training program that included 45 minutes of traditional overground gait training, 45 minutes of activities of daily living and reaching activities in occupational therapy, 45 minutes of biomechanical training in both the upper and lower limbs, 30 minutes of speech therapy, and 30 minutes of rest distributed between sessions (for a total of 195 minutes). When the patients were given the training, they were all in a clinically ON phase (as defined by the UPDRS).

### 2.2 Intervention

GT3 is a platform that combines treadmill gait training with RAS. The device does have an instrumented deck that gives out auditory cues to detect the exact tempo and rhythm during gait training, as well as visual real-time biofeedback to encourage patients to stick to their walking pattern. In fact, the device gives online feedback, such as step length, pace, and symmetry, to motivate patients and track their development. Specifically, the patient had to walk (i.e., to exercise with the gait) by achieving "synchronization" with the RAS pace. Furthermore, the song's beat was enhanced with an overlaid conspicuous high-pitch bell sound, and the song's words were shown alongside it. The patients were initially taught to synchronize their footfall to the beat of the music ("Animals Everywhere"; The Center for Music Therapy, Inc.; Austin, TX, US) that was tailored to their baseline, overground gait performance; that is, the bpm of the soundtrack (RAS's beat frequency, i.e., the music's beat rate) was set considering patients’ cadence measured at the beginning of the rehabilitation program while freely walking overground (mean±sd 85±15 bpm, range 45-105). Then, throughout the course of the first three to five sessions, the beat frequency was gradually increased up to the maximum tolerable bpm (105±12bpm on average, range 80-123). Particularly, the pace was increased in 10% increments every three minutes and according to patients’ compliance, starting from the baseline value (mean±sd 85±15 bpm, range 45-105). The frequency achieved at the fifth session (i.e., 105±12bpm on average, range 80-123) was then used for the remaining part of the RAS training. In other words, the bpm value was kept if the patient was able to maintain step length symmetry safely; otherwise, the training was conducted using the same velocity as in the previous session. The interval between one beat/step and the subsequent one was kept constant in each session. The progression of intensity of training was individually adapted in order to prevent fatigue, for which patients were carefully monitored; in this case, walking speed was reduced to a comfortable pace. Furthermore, heart rate and pulse oximetry were monitored during each session. The gait training frequency was also established by comparing patients’ footfalls in real time to the intended footfalls, and a histogram was created. We chose this target frequency selection and RAS arrangement because it has been demonstrated that employing a beat frequency that is not based on the patient's baseline cadence might affect step length and gait cadence, particularly when the frequency is set too low (60-90bpm) or too high (>150bpm) [16].

### 2.3 Outcomes

Patients were assessed before starting the training and after the end of the training (i.e., the same day as the trial was completed) with regard to the rehabilitation training using FGA, UPDRS, BBS, FES, 10MWT, TUG, and GQI as clinical outcome measures. The physiotherapist who took care of the patient made the measurement twice (with a 5-min break between the measurements) and carefully instructed and supervised the patient concerning measurements. The average score was taken and analyzed. Furthermore, they underwent EEG recording while walking on the treadmill once the fully adapted to the rehab training (i.e., when the target gait was reached in, on average, the third-fifth session) and the last day of training, usually 5-10min after the session started. Specifically, we sought the cerebellum's temporal dynamics by assessing EEG sources resulting from gait, presupposing such task-related cerebellar activity as spatially and temporally distinct from the source activity measured in sensorimotor cortices.

### 2.4 EEG recording and analysis

EEG was recorded using a Brain-Quick System (Micromed; Mogliano Veneto, Italy) equipped with a standard 21-electrode headset (Fp1,Fp2,F7,F3,Fz,F4,F8,T3,C3,Cz,C4, T4,T5,P3,Pz,P4,T6,O1,O2, plus a reference and a ground electrode). EEG was sampled at 512Hz, band-pass filtered at 1-200Hz using a zero-phase finite impulse response(FIR) filter(order=7500) to minimize drifts and a zero-phase FIR filter(order=36), referenced to a common average(for the purpose of source imaging analysis), and notch-filtered at 50Hz(FIR notch filter, order=3302) to remove the power line noise. Impedances were constantly kept below 5kΩ for the entire duration of the experiment and data collection. An electro-oculogram (EOG) with a bipolar montage was also collected. EEG was recorded during the entire gait practice at a full step pace (i.e.,20 minutes), in order to obtain as many trials as possible, because signal to noise ratio in cerebellar EEG recordings is a challenge due to the distance of electrodes to neural signal.

Continuous data were segmented into epochs starting from the right heel strike(HS) and ending at the next one to capture a complete stride(composed of the following, in order: right, left, and right HS).We thus obtained 850±75 epochs. Gait cycle phases were indexed by a wireless inertial sensor (GSensor, BTS Bioengineering; Milan, Italy) fixed on the patient's torso using Velcro straps and used to trigger the EEG. Then, data processed using EEGLab to remove artefactual epochs and components(by visual inspection and Infomax algorithm Independent Component Analysis -ICA) [1].The pruned, segmented EEG (650±80 epochs) was thus submitted to the realignment of the time-points of the epochs for the HS, being the right, left, and right HS time-warped to 0, 50%, and 100% of the gait cycle, respectively [2]. Overall, we took into account the following gait cycle quartiles, which correspond to critical points of gait cycle by a rehabilitative point of view: HS 0%, midstance 25%, end of terminal stance 50%, mid-swing 75%, and the next HS 100% [2].

Source imaging analysis was performed on the epoched EEG using eLORETA plugin into EEGLab and in analogy to previous works on cerebellar EEG [17], [18], [19], [20].The head model for source imaging analysis across all subjects was based on the standard Montreal Neurological Institute coordinates(MNI)-152 template anatomy, adding the cerebellar parcellations in nonlinear MNI-152 space, and finally merging the cortical and cerebellar surface models [21,22].The current dipoles were located at the vertices of the cerebellar and cortical surface models and constrained to orientations orthogonal to their respective surfaces, thus achieving an increased spatial focus in source localization. Source imaging was performed using a weighted-minimum norm estimate applied to the combined cortex and cerebellum model. Specifically, the solution was computed during the entire gait cycle (namely the active period) and referred to as a reference period preceding the gait cycle (−1s before right HS).Finally, voxels and the recording array(electrodes) were co-registered with the Collins 27 MRI by MNI [23]. The Boundary Element Model(BEM) was used for solving the forward problem. We got the Talairach coordinates for each voxel by merging Talairach markers onto the Collins anatomical template [5]. The final coordinates (x, y, z) of the maximal values were based on the Talairach atlas to label the corresponding ROIs. The gait-relevant cortical ROIs were thus re-defined in left and right frontal, central, parietal, temporal, and occipital ROIs (corresponding to sensorimotor affordance, P3/4 and T3/4; motor execution, C3/4, Cz, and FCz; and motor planning, F3/4, Fz, and FCz) [24], [25], [26], whereas gait-relevant left and right cerebellar ROIs were defined on the cerebellum largest activation peaks. The time course of the gait-related activity within each ROI was characterized using the maximum amplitude vertex across the gait cycles. To this end, the current dipole amplitudes were vertex-wise normalized relatively to the baseline, and used to generate source maps with z-score values for each 25% of the gait cycle and subject, and thus averaged across subjects. We used paired t-test for the LORETA solutions to compare the active versus reference conditions in each subject (being the null hypothesis the absence of active vs. reference difference, i.e., the distribution of the voxel values of the participants’ difference inverse solution images has a zero mean). We rejected the null hypothesis for any voxel (no active vs. reference difference) whether the t-values of the un-permuted threshold image was greater than the 95th percentile of the permutation distribution of the maximal statistics [27].

As additional analyses, we carried out a phase-synchronization analysis using the imaginary part of coherency (ImCoh) for detecting gait-induced functional connectivity between O1/O2 and the whole EEG sensor activity. ImCoh has been shown to exhibit the best performance for detecting stimulus-induced functional connectivity changes [28]. Furthermore, we conducted a spherical spline interpolation(SSI) [29], in which the electrodes were projected onto a unit sphere, and a mapping matrix regarding N (O1/O2) and M (other) channels was computed and used to compute interpolated data in the O1/O2 channels. In this regard, we first computed the time-varying, complex-energy, time-frequency distribution (TFD) on consecutive, 2-sec EEG epochs. We then computed the interregional phase locking values (PLV) [30] in terms of consistency of TFD phase differences between O1 and O2(namely, the reference electrodes) with all other near and distant pair-electrodes across the epochs, using original reference-electrode signals and the interpolated signal computed by SSI. The PLV differences were calculated as subtraction between the original and interpolated signals.

### 2.5 Statistical analysis

The Kolmogorov-Smirnov test of normality was used to determine data normality (all p<0.05). Therefore, non-parametric tests were used to compare clinical and electrophysiological measure changes before and after the end of the training at within-group (Wilcoxon test) and between-group level (Mann-Whitney U-test). Multiple Correlation Coefficient was calculated to correlate clinical and electrophysiological changes. To account for multiple comparisons, we corrected significance level as per the Benjamini-Hochberg method. The experimenters and those who analyzed the data (different from the first experimenters) were blind to patients’ group allocation.

## Ethics Statements

Ethical approval: All procedures performed in studies involving human participants were in accordance with the ethical standards of the institutional and/or national research committee and with the 1964 Helsinki declaration and its later amendments or comparable ethical standards. The Institutional Review Board of IRCCS Centro Neurolesi Bonino Pulejo (Messina, Italy) approved the trial.

Informed consent: persons provided their written informed consent to trial participation and data publication.

## Funding

This research did not receive any specific grant from funding agencies in the public, commercial, or not-for-profit sectors.

## CRediT authorship contribution statement

**Antonino Naro:** Conceptualization, Methodology, Writing – original draft. **Loris Pignolo:** Data curation, Software. **Daniele Bruschetta:** Visualization, Investigation, Supervision. **Rocco Salvatore Calabrò:** Writing – review & editing.

## Declaration of Competing Interest

The authors declare that they have no known competing financial interests or personal relationships that could have appeared to influence the work reported in this paper.

## Data Availability

Clinical data repository (Original data) (Mendeley Data). EEGdata repository (Original data) (Mendeley Data).
